# Laser Polishing of Additive Manufactured 316L Stainless Steel Synthesized by Selective Laser Melting

**DOI:** 10.3390/ma12060991

**Published:** 2019-03-26

**Authors:** Muhannad A. Obeidi, Eanna McCarthy, Barry O’Connell, Inam Ul Ahad, Dermot Brabazon

**Affiliations:** 1 School of Mechanical & Manufacturing Engineering, Dublin City University, D09 V209 Dublin, Ireland; eanna.mccarthy@dcu.ie (E.M.); inamUl.Ahad@dcu.ie (I.U.A.); dermot.brabazon@dcu.ie (D.B.); 2 Advanced Processing Technology Research Centre APT, Dublin City University, D09 V209 Dublin, Ireland; 3 I-Form Advanced Manufacturing Research Centre, Dublin City University, D09 V209 Dublin, Ireland; 4 Nano Research Facility, Dublin City University, D09 V209 Dublin, Ireland; barry.oconnell@dcu.ie

**Keywords:** laser polishing, additive manufacturing (AM), metal 3D printing, 316L, stainless steel, surface processing

## Abstract

One of the established limitations of metal additive manufacturing (AM) methods, such as selective laser melting (SLM), is the resulting rough surface finish. Laser polishing is one method that can be used to achieve an improved surface finish on AM printed parts. This study is focused on the laser surface polishing of AM parts using CO_2_ laser beam irradiation. Despite the fact that several researchers have investigated the traditional abrasive polishing method, there is still a lack of information reporting on the laser surface polishing of metal parts. In this study, AM 316L stainless steel cylindrical samples were polished using CO_2_ laser beam irradiation in continuous wave (CW) working mode. Two design of experiment models were developed for the optimization of the input processing parameters by statistical analysis of their effect on the resulting roughness. The processing parameters investigated were the laser beam power, the rotational speed of the sample, the number of laser scan passes, the laser beam focal position, and the percentage overlap of the laser tracks between consecutive passes. The characterization of the measured roughness and the modified layer microstructure was carried out using 3D optical and scanning electron microscopy (SEM). A maximum reduction of the roughness from 10.4 to 2.7 µm was achieved and no significant change in the microstructure phase type and micro-hardness was observed.

## 1. Introduction

Additive manufacturing (AM) is becoming well established in industry for rapid prototyping and manufacturing of parts. AM techniques are becoming increasingly popular in manufacturing industries and are proving very successful in aerospace, automotive, tooling, and biomedical applications. Metals are processed with different processes, such as direct energy deposition and selective laser melting (SLM), also known as powder bed fusion (PBF). The heat source used in these techniques is a laser beam or electron beam. SLM is one of the most important processes used in research and industry, with significant capability in the manufacture of parts with exceptional properties and geometric complexity [1,2,3,4,5,6,7,8]. Compared with conventional subtractive manufacturing methods like Computerized Numerical Control (CNC) milling, turning, and forging, AM can reduce material wastage and production time for specific component designs [9,10]. 

One of the limitations of SLM is the rough finish of the printed parts, requiring manual post-processing to achieve the required roughness. The final roughness depends on many factors such as the initial powder particle size, the building layer thickness, the laser beam power, and the laser scanning speed. The resulting roughness of any AM part is also affected by two main processes—firstly, the well know phenomenon called waving or the stair effect caused by the building the consecutive layers; and secondly, the sintering and adherence of the adjacent not fully fused powder particles on the surface of the produced part, which is called the Balling effect [11,12]. The resulting high roughness of the AM parts limits the ease of employment of the SLM process for high-end applications such as in biomedical implants, in which a non-optimized roughness may create bacterial growth or tissue damage [13], and for high pressure hydraulic valves used in the aerospace industry, where leak tightness is required between connecting components. In aerospace applications, parts also require extremely low roughness for better control over the dimensional accuracy, provision of low friction, and the reduction of surface crack initiation due to vibration and cyclic loads [14]. 

The polishing of AM parts using a conventional method such as abrasive, mechanical, or electro-polishing does not provide an ideal solution because of the lack of dimensional accuracy, especially when selective places require different treatment. Also, electro-polishing has been shown in a number of cases to have a negative impact on the environment [15,16,17,18,19]. There is a similar consideration of the negative effect on the environment during the application of the chemical mechanical polishing technique (CMP), in which chemical and abrasive materials are used [20,21]. AlMangour B. et al. [22] investigated the improvement of the surface quality and mechanical properties of 17-4 stainless steel flat samples manufactured by direct metal laser sintering (DMLS). The researcher used the shot peening technique to reduce the roughness by approximately 70% with a noticeable enhancement in the wear resistance and hardness to the generation of high compressive stresses and grain refinement. 

During recent years, interesting results have been obtained from laser polishing of different metals and non-metal materials. Laser polishing offers an ecologically-friendly method of achieving good finishes without compromising dimensional accuracy. It has the potential to be automated, and even integrated into the production process to allow additive manufacture of useable parts without the need for labour-intensive post-processing. Laser polishing offers flexibility in producing bespoke high levels of surface polishing, as well as high dimensional accuracy. Several researchers have investigated the most significant processing parameters on the final roughness and the resulting chemical and mechanical properties for different metals and metal alloys [23,24,25,26,27,28,29,30,31]. These parameters include the laser beam power, the scanning speed, the laser beam focal position, and the number of scanning passes. The process melts a controlled, very thin localized region from the raised peaks of the part surface, which is then relocated to the valleys of the surface profile, thereby reducing the overall roughness [32,33]. Different surface profiles can be processed once the surface geometry is defined and entered into the laser machine program. Moreover, selective processing can be carried out at discrete locations in order to optimize processing speed and the resulting roughness. Without special optics, however, the laser polishing of the internal surfaces is difficult. Also, in order to achieve symmetrical and consistent Ra, the different geometries and alignments must be treated in a similar manner in order not to be influenced unduly by the effect of gravity or the assist gas. Li et al. [33] investigated the laser polishing of Ti-6Al-4V parts made using the selective laser melting (SLM) technology and achieved a reduction in the roughness from 6.53 to 0.32 microns. The researcher indicated an improvement in the surface mechanical properties such as the micro-hardness and the wear resistance. The laser polishing of different metals has been investigated by several researchers in order to produce a better understanding of the effect of the input processing parameters and to relate them to the modified surface characteristics [34,35]. In order to fill the gap of information available for 316L stainless steel, this paper presents a study of the effect of laser polishing parameters of 316L stainless steel alloy on the resulting surface properties. 

## 2. Materials and Methods

### 2.1. Materials

Firstly, 316L stainless steel samples with cylindrical geometry were additively manufactured and supplied by 3D Systems, High Wycombe, UK, using an EOS M270 3D metal printer made by EOS, Munich, Germany. The initial powder particle size distribution was 15–45 µm, the fiber laser power was set to 195 W with a 1070 nm wavelength, a 0.1 mm spot size, and scanning speed of 750 mm/s. The samples were printed in the vertical direction along the main axis with a layer thickness of 40 µm to the final cylinder diameter of 10 mm and length of 60 mm. Table 1 lists the chemical composition of the stainless steel powder based on weight percentage.

### 2.2. Experimental Set-Up

**Figure 1:**
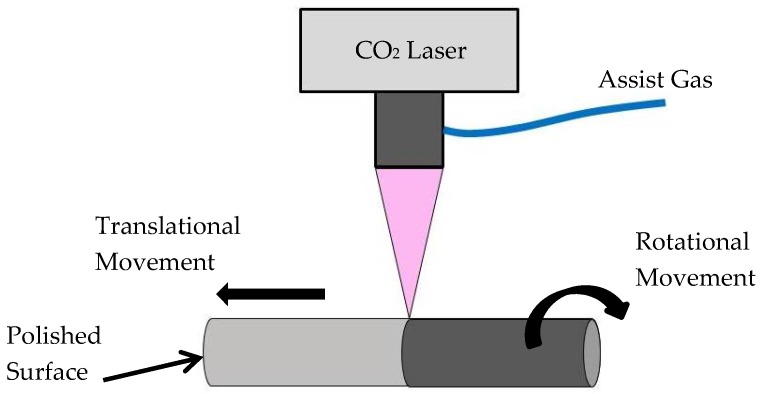
Schematic diagram for the CO_2_ laser polishing scan process of additive manufacturing (AM) produced stainless steel 316L cylindrical samples.

The laser polishing experimental set-up is shown in Figure 1. The laser used in this study was a 1.5 kW CO_2_ laser from Rofin, with a laser beam focus diameter of 0.2 mm. The experimental set-up and the overlapping scanning strategy employed were similar to that described by Obeidi et al. [36,37]. Cylindrical samples were employed in this study instead of flat samples, as with this set-up, this allows for a high range of scanning speeds. The samples were rotated by means of a variable speed DC motor from Bodine Electric company, Chicago, IL, USA, which provided a range of rotational speed of 0–5000 rpm, while the sample and the DC motor assembly were carried on the machine positioning stage, which provided a translational speed range of 0–5000 mm/min. The combination of the set rotational and translational speeds allows the fixed laser beam to scan the entire sample surface in a spiral track with the ability to define the extent of overlap between each track. Argon gas was continuously supplied in the coaxial direction, in-line with the CO_2_ laser beam at a pressure of 50 kPa and flow rate of 30 liters per min in order to provide inert gas surrounding and avoid oxidation.

Carbon dioxide laser was used in this study because it is commonly used in industry owing to the high output power of 0.1 to 50 kW, which can compensate the poor laser–material interaction caused by the long wavelength. Despite the fact that other types of lasers like ND: YAG (neodymium-doped yttrium aluminum garnet) and fiber lasers are also expanding in different industrial applications, the CO_2_ laser is more reliable for the lower initial cost. 

### 2.3. Method

**Figure 2:**
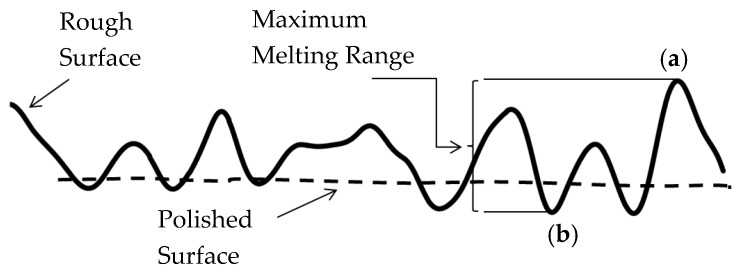
The principle used in the laser polishing process by melting the metal between (**a**) and (**b**) [37].

A preliminary test was used to identify the most significant processing parameters and their levels, with a view of keeping the process just above the melting point in order to avoid any ablation of the material or over melting. Figure 2 shows a schematic diagram for understanding the re-melting process in which the power density and the residence time are adjusted to melt only the high peaks of the material. The roughness peaks, for example, at position (a) in Figure 2, would then be melted and the molten material would move and re-solidify within the lower valleys, for example, at position (b). 

**Figure 3:**
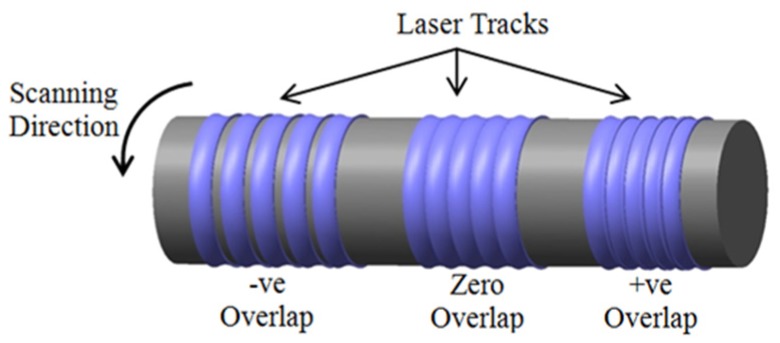
The three overlapping scenarios used for surface laser scanning passes, determined by setting the rate of longitudinal translation of the laser beam.

The experimental work was divided into two design of experiment (DoE) models based on the Box Behnken design. In the first model, the process parameters investigated were the laser power (W) in continuous mode, rotational speed (rpm), and the number of repeated passes. In the second model, the optimum values of the laser beam power (110 W) and the number of repeated passes (one) were adopted from the first model. The rotational speed was also examined in the second module, as well as two new parameters, the focal position (below, on, and above the sample surface) and the percentage overlap of the laser spot in the axial direction, as shown in Figure 3. Table 2 lists the processing parameters and their levels investigated. No significant effect for the rotational speed was noted in the results of DoE-1 compared with the other two parameters in DoE-1. The speed parameter was thus expanded to a testing range between 20 to 80 rpm in DoE-2.

The percentage overlap processing parameter here means the percentage of the beam spot diameter that goes over the previous laser track that has already been processed [16]. Three possible overlapping scenarios were applied: negative, zero, and positive overlapping laser tracks. Here, the negative overlap indicates unprocessed gaps between the consecutive passes; conversely, the positive overlap means the laser tracks interfere with each other; and the zero overlap indicates that the laser tracks just touch each other tangentially—see Figure 3.

The roughness profile was measured by means of non-contact, 3D microscope, from Keyence VHX2000E 3D digital, Milton Keyense, UK and Bruker Contour GT-X Profilometer (Billerica, MA, USA). At least five surface roughness profiles were obtained for each sample in the DoE parallel to the cylinder axis for a length of 4.75 mm and the average surface roughness was calculated from these profiles along with the 95% confidence intervals (CI). The initial average roughness, Ra, for the as-built samples was measured at 10.4 µm. 

The cross-sectional microstructure was also investigated for as-received and laser-processed metal additive manufactured samples in order to compare the effects of the sample processing. The samples were sliced using a diamond disc cutter of 0.5 mm thickness and were polished by removing a layer thickness of no less than 0.5 mm in order to avoid the effect of the cutting process on the microstructure. Silicon carbide papers with different grades of 400, 600, 800, and 1200 were used for the grinding with continuous water stream for flushing the loose and abrasive particles, using a Metkon Forcimat grinder-polisher, Bursa, Turkey. The polishing process was conducted using diamond suspension with 9, 6, 3, and 0.05 micron particle size on a Struers Textmet cloth from MetPrep, Coventry, UK. Each polishing grade was applied for three minutes at a rotational speed of 300 rpm. 

The effect of the laser process on the sample hardness was also measured in the cross-sectional direction. The aforementioned grinding and polishing process was applied to remove no less than a 0.5 mm layer in order to avoid the effect of slicing and to guarantee the removal of any residual stresses. Leitz Wetzlar Germany Vickers micro-hardness tester (Model: 301-252.001, Wetzlar, D-35578, Germany was used to measure the hardness of the as-built and the laser processed samples in the cross-sectional direction. The measurements start from the sample surface and move toward the center in three parallel lines in order to compare the hardness of the melted and re-solidified layer with the unprocessed bulk material. 

## 3. Results

The average roughness, Ra, of the as-built metal AM samples was found to be 10.4 µm, and the arithmetical mean height (S_a_) was 25 µm. The maximum and the average of maximum height of the profile Rt and Rz are equal in this case because one single roughness profile of 4.75 mm was measured in five different locations and averaged (µm). For the as-built samples, this value was found to be 38.77 µm.

**Figure 4:**
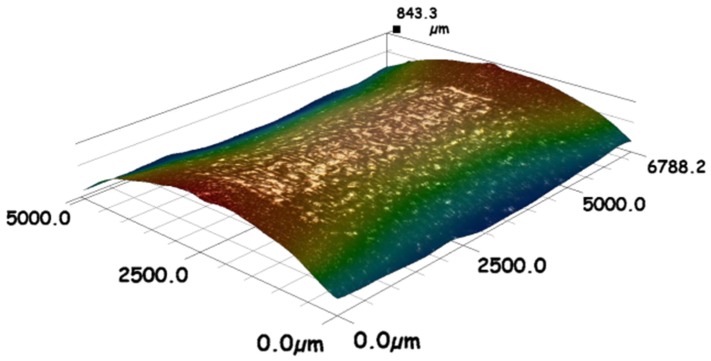
Three-dimensional (3D) microscope image of 316L SST AM sample after CO_2_ laser polishing with 110 W, 20% percentage overlapping laser scans (OV), 20 rpm, and laser beam focused on the surface.

**Figure 5:**
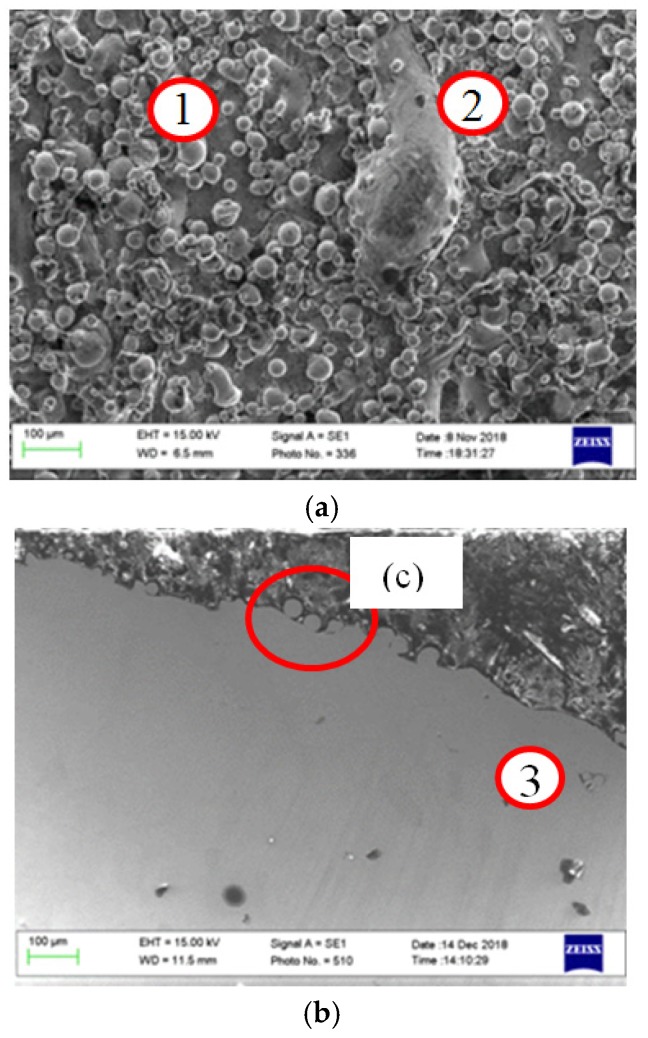

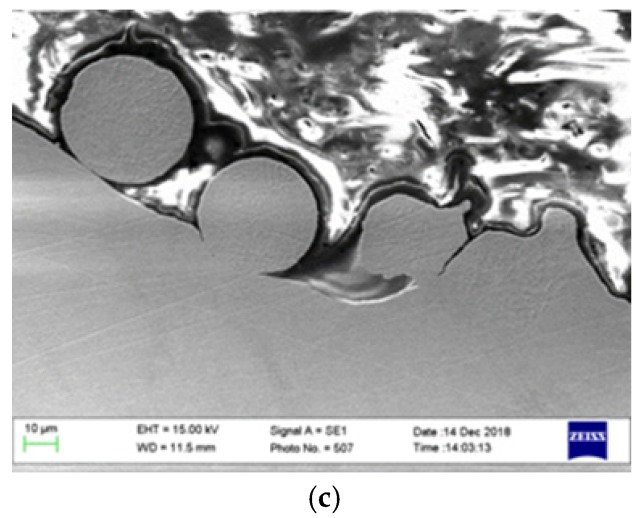
(**a**) Scanning electron microscopy (SEM) micro-image of the metal AM sample surface showing (**a**) the surface morphology with the fully melted material in the back ground, zones (1) and (2) show the BALLING effect and the partially melted and adhered neighboring particles, (**b**) shows the cross-section SEM micrograph view of the same surface, zone (3) shows unmelted powder particles, and **(c**) shows a high magnification from the micrograph shown in (**b**).

As mentioned previously, the roughness profile is constructed from, firstly, the topography caused by the melting of the consecutive layers and, secondly, the adhesion of adjacent and partially melted powder particles at the boundaries. Figure 4 shows a 3D microscope image of the AM sample after laser polishing of 5 mm length along the main axis of the cylindrical sample. The polished sample was processed with 110 W, 20 rpm, 20% percentage overlapping laser scans (OV), and laser beam focus on the surface. The scale on the z-axis also includes the curvature of the cylindrical geometry of the sample. Figure 5a–c show scanning electron microscopy SEM images taken by means of (Model: EVO LS 15-0723 from Carl Zeiss Ltd., Cambridge, UK) microscope, for the surface morphology and the cross-sectional view. In this figure, zones (1) and (2) show the rough surface of the as-built parts, which is mainly caused by the sintering of the adjacent powder particles in the balling effect, which is similar to micro-welding. Zone (3) shows voids in the build part represented by unfused powder particles, which could be because of a lack of fusion. 

**Figure 6:**
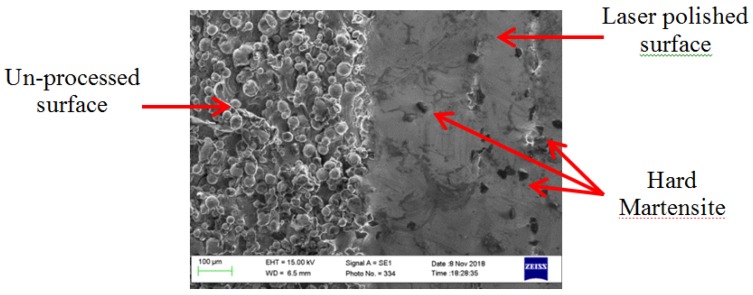
SEM image of AM 316L SST sample showing the same sample surface before (left) and after (right) laser polishing with 130 W, 30 rpm, and two passes repetition.

The processed samples exhibit a wide range of variation of the roughness depending on the input parameters. The presence of the hard martensitic isolated islands was observed on the re-solidified surface surrounded by the soft austenite, as can be seen in Figure 6. The measured micro-hardness was found to be ~234 HV on the as-built and processed samples. 

No significant change in the micro-hardness of the polished samples was observed despite the fact of the hard martensite formation. This can be explained by the small amount of the hard phase formed and surrounded by the soft austenite phase, which can absorb the hardness tester probe impression and show no alteration in the hardness—see Figure 6. This result agrees well with the results achieved by Obeidi et al. [26] and Kato et al. [38]. 

**Figure 7:**
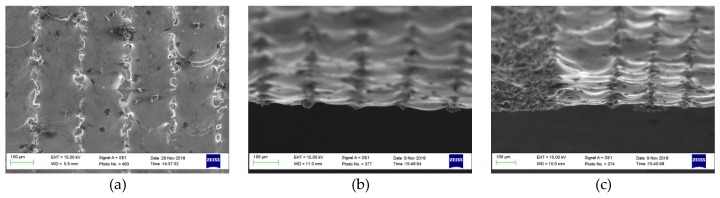

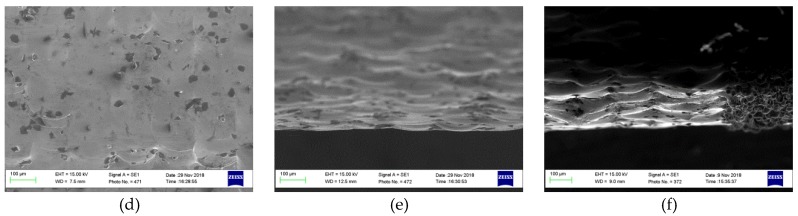
SEM micrograph showing the top and side views of AM 316L SST samples polished with 50 rpm, 0% overlap, one scanning pass, and 90 W in (**a**) top, (**b**), and (**c**) side-view, as well as 130 W in (**d**) top, (**e**), and (**f**) side view.

Figure 7a shows the plan view (top down) and Figure 7b,c show the side-view SEM images for a sample processed with 50 rpm, 0% overlap, one pass, and 90 W. Figure 7d shows the plan view and Figure 7c,f show the side-view SEM images for a sample processed at 50 rpm, 0% overlap, one pass, and 130 W. Because of the Gaussian energy distribution of the CO_2_ laser beam in the cross-sectional direction, the samples show unmelted gaps between the laser passes when the lower power level of 90 W was employed, as shown in Figure 7a–c. Conversely, when the higher power level of 130 W was applied, a clear interference of the melt-pools can be observed—see Figure 7d,c,f.

**Figure 8:**
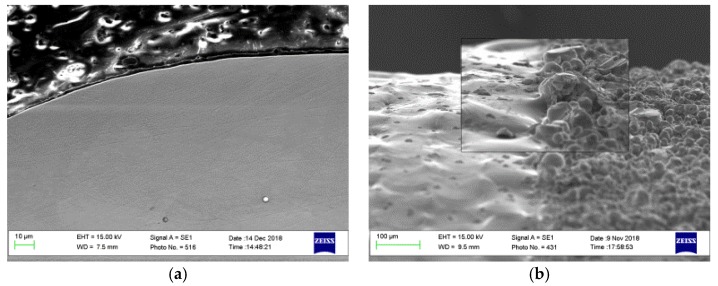
SEM micrograph shows (**a**) the cross-sectional view of the polished sample and (**b**) the side view. This sample was processed with 110 W, 20% overlap, and 20 rpm, and the laser beam was focused on the sample surface.

In the cross-sectional direction, the melted and re-solidified layer thickness was found to vary from 10 to 80 µm. Figure 8a shows the peripheral external edge of the sample in which most of the surface undulations and the adhered powder particles present in Figure 5b were removed. Because of the polishing process, a reduction in the sample diameter from 10.0 ± 0.015 mm to 9.97 ± 0.006 mm was measured. The resulting reduction in sample height after polishing compared with before can be seen in Figure 8b. 

**Figure 9:**
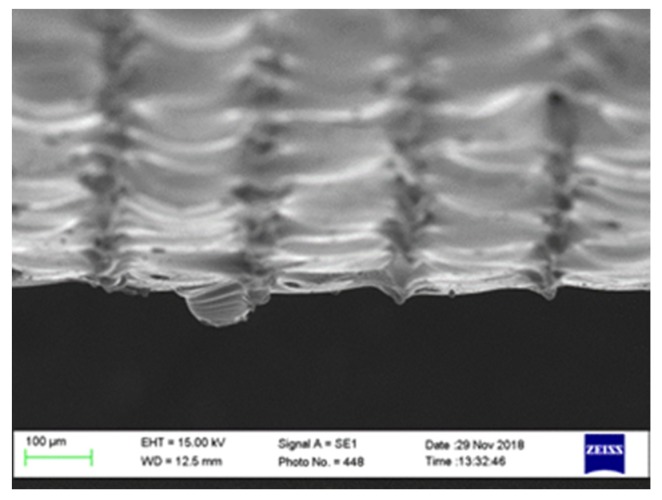
SEM image of a remaining unmelted particle due to lack of fusion.

Figure 9 shows an unmelted particle on the polished sample surface, which results from an inconsistent surface similar to that in Figure 5a zone (2).

**Figure 10:**
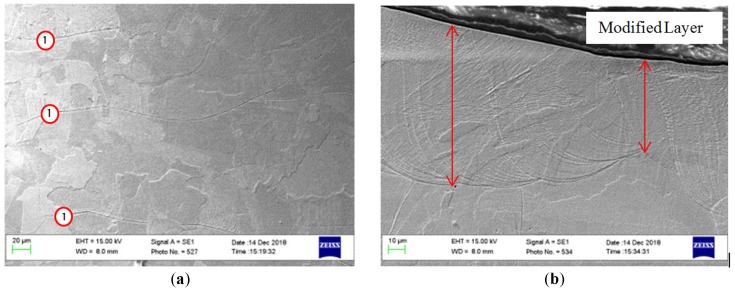
SEM micrograph of the cross-section of the (**a**) as-received and (**b**) re-melted modified layer of the polished sample processed with 110 W, 20 rpm, 20% OV, and the laser beam focal positioned on the sample surface.

Figure 10a shows the austenitic microstructure of the bulk material and the build layers boundaries for the AM sample. Figure 10b shows the modified layer in the polished sample. The microstructure of 316L SST is austenite at room temperature, and the large grains, can grow even through the different melt-pool boundaries, as shown in area (1) in Figure 10a.

**Figure 11:**
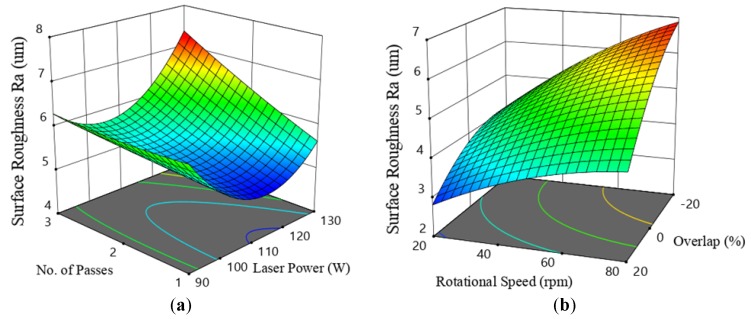
Response surface model (RSM) graphs (**a**) related to design of experiment (DoE)-1 in Table 2, showing the interaction effect on Ra of the laser beam power and the number of passes at 50 rpm; and (**b**) related to DoE-2, the effects of rotational scanning speed and the percentage overlap, with the focal position set on the sample surface.

The response surface model (RSM) graphs obtained from DoE-1 show that the lowest roughness values were always obtained when using the laser beam power of 110 W with one scanning pass—see Figure 11a. In the DoE-2 test, the laser beam power and the number of passes were kept constants at 110 W and one pass, respectively, and two new parameters employed were the percentage overlap and the laser beam focal position. A noticeable improvement was found in the produced roughness. Predominantly, the positive overlap, when applied in combination with the lower rotational speed level, results in a low Ra. No significant effect for the focal position was observed for the range applied. Lower roughness was also found at the lowest level of rotation speed.

The surface profile was investigated as formerly explained in one cut-off with the measured length of 4.75 mm. Table 3 lists the processing parameters applied with the resulting measured average roughness R_a_ and the average maximum height R_z_, which is equal to the maximum height of the profile in this case of one cut-off profile.

**Figure 12:**
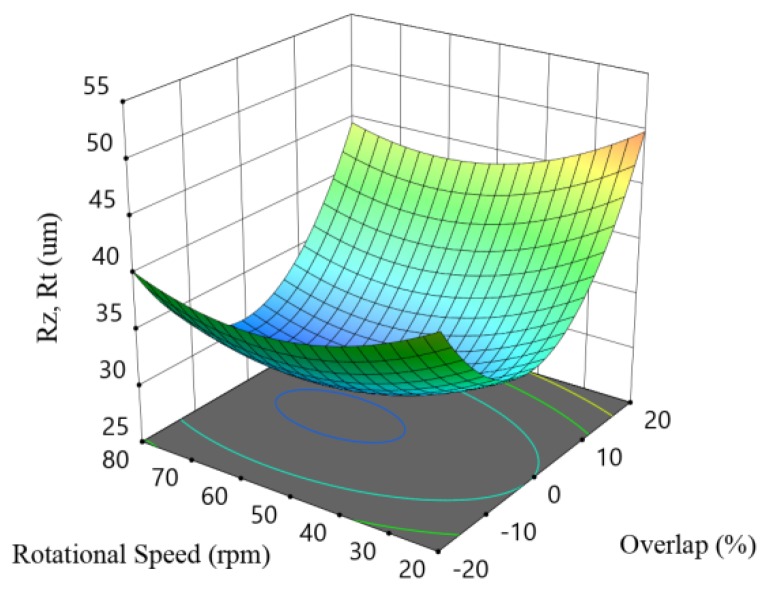
RSM plot correlating the R_z_ with the processing parameters in DoE-2.

Figure 12 shows the response surface method (RSM) plot for R_t_ and R_z_ with the overlapping percentage and the rotational speed corresponding to the data listed in Table 3 when the laser beam focal is positioned on the sample surface. 

**Figure 13:**
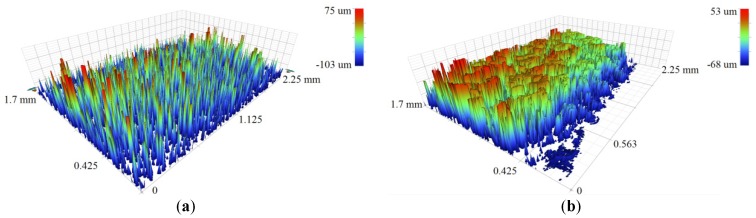
Three-dimensional (3D) optical images of the surface topography of (**a**) as-built and (**b**) laser polished samples.

Figure 13 shows the optical micrographs extracted by Bruker Contour GT-X for the surface topography of the as-built and a laser polished sample processed with 20% OV, 20 rpm, and the laser beam focal positioned on the sample surface. The surface analysis of these data shows a noticeable reduction in the arithmetical mean height (S_a_) after laser polishing. The measured value of (S_a_) was reduced from 25 µm for the as-built to 11 µm after laser polishing. 

## 4. Discussion

It is mentioned previously that depositing the consecutive metal layers on a curvature or inclination of geometry will results in increasing the roughness as a result of the Stair Steps effect in addition to the roughness caused by the sintered neighboring partially fused powder particles. Meanwhile, printing in the vertical direction minimizes the stair steps effect on the surface roughness, which is simply similar to the waving surface. Figure 15 below shows both printing scenarios. 

**Figure 14:**
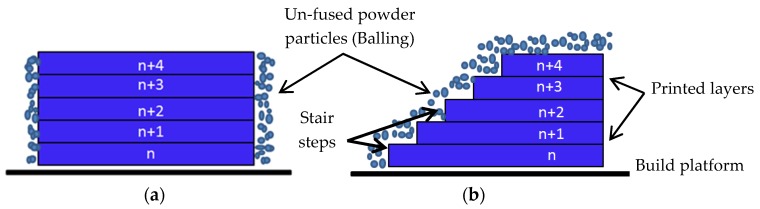
(**a**) Printing in the vertical direction and (**b**) printing curvature geometry.

In this study, the AM samples were built in the vertical direction, as shown in Figure 14a, so the waving effect on the surface was limited and thermal energy was mainly applied to melt the partially fused particles adhered on the surface. Laser polishing of parts with curved geometry, shown in Figure 14b, might require a small amount of thermal energy, higher than that in Figure 14a, or may require multi-passes of laser beam scan because the process is in the micro-scale of powder particles and layer thickness. 

The applied thermal energy was adjusted to be just at the melting threshold—firstly, in order to avoid the loss of the material; and secondly, to avoid the undesired overmelting of the bulk material [30]. The presence of some pores, voids, and unmelted particles in the AM samples, as shown in Figure 5b zone (3), can be explained by the lack of fusion and the trapping of gases during the re-solidification. A significant reduction in the mechanical properties, due to the ease of crack initiation, can be expected as a result of these surface voids.

**Figure 15:**
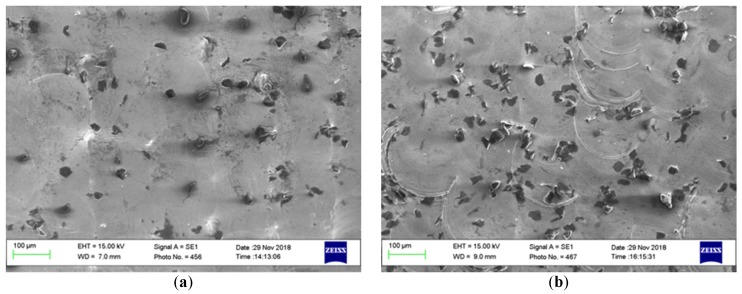
SEM micrograph showing the martensitic islands on the top view of samples processed with 110 W, beam focused on surface, and (**a**) 50 rpm and (**b**) 80 rpm.

The microstructure of 316L SST is austenite at room temperature. It was noted that the modified layer in the polished samples exhibits the same microstructure with full chemical and mechanical bonding with the bulk material. The presence of some lamellar grains of the hard martensitic laths was also noted on the surface due to the high cooling rates in this region, enhanced by the flow of the assist argon gas over the small melt pools, as shown in Figure 15. This figure shows SEM micrographs for the top view of samples no. 5 and 8 corresponding to Table 3 respectively. The samples exhibit the minimum and maximum amount of martensite formed on the surface after the laser process. Image-j software (version V1.52a) was used to calculate the transition rate, which was found to be 2% and 3.1%, respectively.

It is clear to see that the amount of martensitic islands is greater in Figure 15b compared with that in Figure 15a because of the higher scanning speed and the resultant higher cooling rates. The higher cooling rate caused by the higher rotational speed in the case of the sample in Figure 15b allows for the increase in the formation of the martensitic laths in the modified layer. 

In laser polishing, the challenge is the accurate and critical adjustment of the laser beam power, scanning speed, and the residence time, which in turn control the applied thermal energy. When these parameters are under control, one laser scan track was sufficient to give the optimum reduction in roughness. For repeated passes, a re-melting of the processed surface and increased roughening occurred—see Figure 11a. 

The Gaussian energy distribution of the CO_2_ laser beam leaves un-melted gaps at the low energy density zones on both sides of the laser beam track. In order to overcome this problem, a positive overlapping scanning of the consecutive scans and/or an increase in process power can resolve this. An increased overlap, however, results in an increase in the overall processing time. The percentage overlap control was found to provide more control over the roughness than control of laser power. 

Figure 11a indicates that the laser power range used was well estimated and that the central level was sufficient to create the required amount of melting. The number of process passes should be kept at one. DoE-2 indicates that the beam focal position that produces the minimum Ra occured when the focus was on the sample surface, in which the highest thermal energy was focused on the elevated peaks and the adhered particles, which leads to the melting of the majority of these peaks, thereby enabled a more effective surface smoothening. Figure 11b indicates that the lower roughness was reached when 20% overlap was applied with the minimum rotational speed of 20 rpm. The lower speed allows for the melting of the high metal peaks and the relocating of this molten material to the depressed areas—see Figure 10b.

The results listed in Table 3 show that although the reduction observed on Ra was significant, there was no improvement in R_t_, R_z_, and S_a_. This can be explained by two main effects. Firstly, the waving presented on the re-solidified layer due to the high convection caused by the argon assist gas flow, as well as the high temperature difference between the molten temperature at the laser incident point and the solidification front. Secondly, the surface topography of the as-built samples is mainly constructed by isolated peaks—see Figure 13a. The major part of these peaks can be melted and relocated in the low valleys, but this may result in high amplitude topography. 

The initial powder particle size distribution was between 15 and 45 µm, and the measured reduction in the sample diameter was found to be from 10.0 ± 0.015 mm to 9.97 ± 0.006 mm (~15 µm in the radial direction), which is less than the smaller particle size. This indicates that the polishing process was carried out by melting the adhered powder particles on the outer surface of the AM parts, and that no material loss occurred. No excessive melting occurred in the bulk material. The resulting roughness variation was also significantly reduced from 15 µm to 6 µm. This is an important benefit in the control and the adjustment of the dimensional accuracy of the mechanical parts manufactured using the AM technology, especially when these parts assembled with other parts where the interfacing area is exposed to interference fit or requires a level of control over fluid containment.

## 5. Conclusions

In this study, the laser polishing of AM stainless steel cylindrical samples was investigated. The optical results show that the polishing strategy used was effective, and achieved a reduction of the average roughness Ra from 10.4 to 2.7 µm. This achievement offers great benefits compared with conventional chemical, mechanical, and electro-polishing, which involve tool wear, abrasive debris, and environmentally damaging solvents. Laser polishing is still under investigation and development, and there is a lack of sufficient data for the optimization of this technique for a broad range of cases. One of the current difficulties remains in defining the exact level of laser beam fluence required, which might be locally different because of surface inconsistencies, as shown in Figure 5a zone (2), where a larger mass would require a higher power density than the surrounding smaller particles or a longer residence time. This problem can result in leaving residual unmelted particles, as shown in Figure 9. As a possible solution to overcome this problem, real-time closed loop optical with pyrometer or image-based monitoring could be used to adjust the fluence during processing.

## Figures and Tables

**Table 1:** The chemical composition of the 316L stainless steel powder.

**Chemical Composition**	Cr	Cu	Fe	Mn	Mo	Ni	P	S	Si	C	O	N
**Content (wt.%)**	17.42	0.02	66.52	0.60	2.36	12.53	0.01	<0.01	0.51	0.02	0.05	0.06

**Table 2:** The laser processing parameters implemented for design of experiment (DoE) model 1 and 2, both according to the Box Behnken model.

**DoE-1**			
Processing parameter	Level 1	Level 2	Level 3
Power (W)	90	110	130
Rotational speed (rpm)	30	50	70
No. of passes	1	2	3
**DoE-2**			
Overlap (%)	−20	0	20
Focal position (mm)	−0.5 (beneath surface)	0 (on surface)	0.5 (above surface)
Rotational speed (rpm)	20	50	80

**Table 3:** The average roughness, R_a_, and the maximum and average height, R_t_ and R_z_, respectively, of the measured roughness profile.

Sample No.	OV	Rotational Speed	F.P	R_a_	R_z_, R_t_
1	0	50	0	5.14	34.05
2	0	80	−0.5	5.04	32.57
3	−20	50	−0.5	6.08	39.38
4	0	50	0	4.98	27.71
5	20	20	0	2.74	47.17
6	0	20	−0.5	4.16	33.36
7	0	50	0	4.99	27.71
8	20	80	0	4.26	39.92
9	0	50	0	5.77	30.45
10	−20	80	0	6.90	38.38
11	20	50	−0.5	4.11	47.44
12	−20	50	0.5	5.44	37.81
13	20	50	0.5	3.47	45.86
14	0	20	0.5	4.43	32.72
15	−20	20	0	3.99	48.28
16	0	50	0	4.82	29.01
17	0	80	0.5	6.03	39.54

where OV is the percentage overlapping laser scans (%); Ra is the average roughness in (µm); Rt and Rz are the maximum and average of maximum height of the profile, respectively, in (µm); the rotational speed is in (rpm); and F.P is the laser beam focal position in (mm). Ra, Rz, and (Rt) were averaged from five profile measurements.
